# Mortality from Breast Cancer in Women under 50 Years of Age in Colombia

**DOI:** 10.1055/s-0043-1775881

**Published:** 2023-12-23

**Authors:** Mario Arturo González Mariño

**Affiliations:** 1 Department of Obstetrics and Gynecology, Faculty of Medicine, Universidad Nacional de Colombia, Bogotá, Colombia

**Keywords:** neoplasms, breast, mortality, women, demography, Colombia, South America

## Abstract

**Objective** To calculate and analyze the mortality rates from breast cancer in women under 50 years of age in Colombia and to compare them with those of other countries in the region.

**Methods** Based on data from the registry of deaths in 2018 and the results of the National Population and Housing Census of Colombia for the same year, specific mortality rates in women with breast cancer, specific mortality according to age group, standardized by age, proportional mortality, potential years of life lost, and years of life expectancy lost in women under 50 years of age who died from breast cancer were calculated. The mortality rate of regional countries was consulted on the Global Cancer Observatory webpage.

**Results** In the group from 20 to 49 years, the specific mortality rate was higher in the age range from 45 to 49 years, with a rate of 23.42 × 100,000, a value that was above the specific mortality rate due to breast cancer in women in Colombia, 15.17 × 100.000. In the age range of 45 to 49 years, the potential years of life lost were 42.16. Of the 0.275 years of life expectancy lost by the population due to this neoplasia, women under 50 years of age represented 0.091 (33%). Colombia is the fifth in the rank of mortality in Latin American countries in this age group.

**Conclusion** Breast cancer in patients from 30 to 59 years is the number one cause for the decrease in life expectancy of women in Colombia. Women under 50 years of age represent one third of this decrease. This neoplasm is also the leading cause of mortality in women younger than 50 years in South America.

## Introduction

The most diagnosed cancer in the world is breast cancer (with an estimated 2.3 million new cases annually), and it is the leading cause of cancer death in women. 1 In women under 50 years of age, this position is maintained, with an estimated of 665,508 new cases and a mortality of 131,322. 2 In low- and middle-income countries, breast cancer patients tend to be younger than in high-income countries. 3 Breast cancer, particularly in women under 40 years of age, has a worse prognosis than in older women, 4 especially luminal A-type tumor (regardless of the state at the time of diagnosis). 5 In these women, the tumors are characterized by a higher proportion of histological grade 3 tumors, triple negative or HER2 overexpression, lymphovascular invasion, and lymphocytic infiltration. Intrinsic basal-like subtypes and HER2-enriched tumors are more frequent. 6 Their detection is usually reported in a more advanced stage 7 although in other studies, young age is not an independent factor of delay in diagnosis. 8 9

Breast cancer at a young age is associated with higher costs in medical treatment, loss of productivity, 10 11 12 labor, and financial problems due to reduced working hours, and in the long term, a higher unemployment rate. 13 Increased risk of psychosocial morbidity after diagnosis has been documented, particularly in those receiving chemotherapy and/or undergoing the menopausal transition with treatment. 3 14 15

Compared with North America and Europe, Latin American women are also diagnosed at a younger age. 16 The rising predicted trend in the 20 to 49 age group is possibly due to changes in menstrual (early menarche) and reproductive factors as well as in lifestyle habits. 16 However, specific risk factors for breast cancer trends in Latin America remain largely unknown. 17

In Colombia, the highest proportion of new cancer cases in the female population corresponds to breast cancer (4,506 new cases), 18 which also has the highest number of cancer deaths, 18 with 3,428 deaths, of which 763 (22.25%) were of women under 50 years of age in 2018, 3 of them between 20 and 24 years as the lower limit. 19 According to the census, the population of women between 20 and 49 years old was 10,007,332 (44.29% of the total number of women). 20

Mortality rates can serve as a measure of disease severity and help determine whether the treatment has become more effective over time. 21 They can be classified in general, as a summary form of the risk of dying in the general population, but since the risk of dying is strongly related to age, to perform a more precise analysis of the risks of dying, specific mortality rates are used. 22 To compare mortality rates considering differences in age distribution between populations or the same population in different periods, standardized or adjusted mortality rates are used. The goal is to eliminate the influence of different age structures on the mortality rates being compared. 22 23 However, care must be taken in the comparison since the data used in its calculation may come from different sources, be estimates, or differ in the selected standard population.

Proportional mortality is a useful measure to describe the relative weight of the various causes of total deaths. It is a frequently used indicator despite its limitations. A change in the proportional mortality of a certain disease over time may be due not to changes in the mortality of that disease, but to changes in the mortality of some other disease. 24

Years of potential life lost (YPLL) refer to the losses suffered by society because of the deaths of young people or premature deaths. It has been increasingly used to set health priorities. This indicator in years is higher the younger the person who died. Its limits vary; the most recommended by developing countries are 0 to 65 years, while developed countries suggest 1 to 70 years. 25

The years of life lost index is derived based on life table functions and is related to the decomposition of changes in life expectancy. 26

The GLOBOCAN, used in this study, is an online database that provides global cancer statistics and estimates of incidence and mortality in 185 countries for 36 types of cancer and all cancer sites combined. 27 The data are part of International Association of Cancer Registries (IARC)'s Global Cancer Observatory, a web-based platform presenting global cancer statistics to inform cancer control and cancer research on cancer. 2

## Methods

This is a descriptive study that collects data on the registry of deaths in 2018, results of the National Population and Housing Census of Colombia in the same year, both reported by the National Administrative Department of Statistics (DANE, in the Spanish acronym) and information from the references cited in the text with which the indicators are constructed and developed. The specific mortality rate due to breast cancer in women, the specific mortality rate due to breast cancer according to age group in women, the standardized rate by age (in groups by age and gender), proportional mortality, potential years of life lost, and years of life expectancy lost (corresponding to the years 2017 to 2019) in women under 50 years of age who died from breast cancer were calculated.

The data for comparison of breast mortality in women under 50 years of age among South American countries were obtained from GLOBOCAN by the Global Cancer Observatory.

Of the 4,506 new cases of breast cancer in Colombia in 2018, there were 1,362 (30.22%) under 50 years of age. The intrinsic subtypes of these tumors have not been well characterized in the country, but in an institutional study, 28 it was found that of 468 patients with breast cancer, 131 cancers were in patients under 50 years of age, with luminal A being the most frequent (30.53%), followed by luminal B Her2 negative (27.48%). Triple-negative cancer was found in 16.79% of women under 50 years of age with breast cancer, but its proportion to the total of its intrinsic subtype was the highest (36.06 of the total triple negatives). In cancers in women 50 years of age or older, the same order is maintained. Overall survival according to age was reported to be 88.4% (95%CI: 85.5–90.8) in women younger than 50 years with a median follow-up of 41 months, and 88.3% (95%CI: 86.3–90.0) with a median follow-up of 40 months in women aged 50 and over. 29

## Results

Colombia's specific mortality rate for breast cancer in women was 15.17 × 100,000. The proportional mortality in women with breast cancer under 50 years of age was 0.71. The specific mortality rates for breast cancer according to the age group, age-standardized rates, and potential years of life lost are presented in Table 1 . Women under 50 years of age with breast cancer contributed 0.091 years of life expectancy lost to the population. The mortality rate from breast cancer standardized by age per 100,000 from 0 to 49 years in the region shows the Bolivarian Republic of Venezuela and Uruguay (5) as the countries with the highest rates followed in descending order by Brazil (4.6), Argentina (4.5), Colombia (3.9), Ecuador (3.3), and Peru (3.1).

**Table 1:** Number of deaths from breast cancer, population of women by age group, specific mortality rates for this pathology by age group, age-standardized rates, and years of potential life lost in Colombia

Age	Population	Deaths	SMR	Weighing*	ASR	YPLL
20–24	1 956 735	3	0.15	0.0867	0.01	0.65
25–29	1 857 016	28	1.50	0.0837	0.12	5.72
30–34	1 700 746	72	4.23	0.0803	0.33	13.97
35–39	1 656 227	143	8.63	0.0755	0.65	24.17
40–44	1 436 336	189	13.15	0.0695	0.91	30.26
45–49	1 400 272	328	23.42	0.0637	1.49	42.16

Abbreviations: ASR, age-standardized rates; SMR, specific mortality rates; YPLL, years of potential life lost.

Weighting* = weighting in the standard population (WHO world standard population).

**Source:** Pan American Health Organization (PAHO) 2009. Age-standardized death rates per population of 100,000. https://www.paho.org/hq/dmdocuments/2010/Tasas-de-mortalidad-por-edad-estandarizadas-hoja-de-resumen.pdf . Accessed 2023 January 15. (Spanish). 23

## Discussion

Mortality is one of the most important indicators for monitoring the health of patients with breast cancer. 30 This neoplasm is the most frequent type of cancer in Latin American countries and the leading cause of cancer mortality among women. The mean age of diagnosis and death from this neoplasia in the region is ∼ 10 years younger than that reported in developed countries (except Argentina and Uruguay). 16

The age-standardized mortality rate in women under 50 years of age in 2008 was reported in Uruguay and Argentina at 7, in Brazil and Ecuador at 14, and 15 × 100,000 in Colombia. 16 For the year 2020, Colombia reported lower mortality rates than Uruguay and Argentina. 2

The mortality from cancer in Latin American countries is approximately 2-fold higher than it is in more developed countries, 31 and the incidence and mortality are likely to continuously increase in the coming decades. 32 It must be considered that developing countries like those in Latin America are subjected to serious problems in access to health services, including those to obtain diagnosis and modern treatments, 33 which contributes to the increased mortality rates from breast cancer. The Organization for Economic Co-operation and Development (OECD) and the World Bank, within the indicators to measure the quality control of medical care, describe the survival rates for breast cancer as a reflection of the quality of preventive and curative care. Among the countries of Latin America and the Caribbean with data for this indicator, women with an early diagnosis of breast cancer had an average 78% probability of surviving at least 5 years. Below this percentage were Ecuador (76%), Brazil (75%), and Colombia (72%), while Argentina (84%) and Peru (82%) exceeded this threshold. 34

The number one cause for the decrease in life expectancy of women in Colombia from the age of 30 to 59 is malignant breast tumors. Of the 0.275 years of life expectancy lost by the population due to this neoplasm in the country, 21 women under 50 years of age contributed one third.

The data reported in this study showed that the age-standardized breast cancer mortality rate in women under 50 years of age in Colombia was lower than that reported in other studies. 2 17 In the case of the publication by Carioli et al., 17 the mortality from breast cancer standardized by age (20–49 years) expected for 2019 was 5.65 (5.13–6.16), while in this study, in 2018, it was 3.52. However, the census data sources used in this study do not allow direct comparison with other reports made with population estimation.

The mortality indicators calculated in this article become important when studies are performed with the same methodology over time. Brazil has mortality trend studies that show that breast cancer mortality in young women has increased in the last 2 decades, 35 with an average increase of 0.18 per year; *p* < 0.001, with regional differences, particularly in the 20 to 49 years age group (0.07 per year; *p* < 0.001). 30 Some studies suggest that this increasing breast cancer incidence in young women occurs in the absence of family clustering, reflecting a change in the distribution patterns for this neoplasm. 36

The limitations of this study in its report have to do with the quality of the information source. Death registries can present flaws in their processing, although this aspect has been improving, with figures that report 92.8% of well-certified cancer mortality. 37 Comparison with other countries is difficult due to different sources of data, estimate-based studies, differing in the standard population selected, reference ages, and different calculations of death indicators in women.

## Conclusion

Mortality is an index of the severity of a disease from a clinical and public health viewpoint. Knowing its indicators is important in planning public policies. Breast cancer is the leading cause of cancer mortality among women in Latin America. The growing mortality in the population of women under 50 years of age should attract the interest of health offices to generate clinical and population awareness of breast cancer in women in this age group.
